# Odontogenic Keratocyst Involving the Maxillary Sinus: A Rare Diagnostic and Therapeutic Challenge

**DOI:** 10.1155/crid/3997118

**Published:** 2026-11-06

**Authors:** Rafaela Nascimento Santana, Lucas Alves da Mota Santana, Gina Delia Roque-Torres, Rajiv Gandhi Gopalsamy, Danyel Elias da Cruz Perez, Janaína Araújo Dantas, Paulo Nand Kumar, Leandro Napier de Souza, Carlos Eduardo Palanch Repeke, Liane Maciel de Almeida Souza

**Affiliations:** ^1^ School of Dentistry Federal University of Sergipe (UFS) Lagarto Sergipe Brazil ufs.br; ^2^ School of Dentistry Federal University of Sergipe (UFS) Aracaju Sergipe Brazil ufs.br; ^3^ Center for Dental Research School of Dentistry Loma Linda University (LLU) Loma Linda California USA; ^4^ Division of Phytochemistry and Drug Design Department of Biosciences Rajagiri College of Social Sciences Kochi Kerala India; ^5^ Department of Clinical and Preventive Dentistry Oral Pathology Unit Federal University of Pernambuco (UFPE) Recife Pernambuco Brazil ufpe.br; ^6^ Department of Oral and Maxillofacial Surgery Urgency Hospital of Sergipe (HUSE) Aracaju Sergipe Brazil; ^7^ Department of Oral Surgery and Pathology School of Dentistry Federal University of Minas Gerais (UFMG) Belo Horizonte Minas Gerais Brazil ufmg.br

**Keywords:** conservative treatment, maxillary sinus, odontogenic keratocyst, panoramic radiography

## Abstract

Odontogenic keratocyst (OKC) is a benign cystic lesion with aggressive behavior and a high recurrence rate. The occurrence of this lesion in the maxillary sinus is extremely uncommon, accounting for approximately 1% of described cases. Here, we report a rare case of OKC involving the maxillary sinus, incidentally diagnosed during surgical evaluation of a third molar in a 24‐year‐old male patient. Panoramic radiography revealed extensive radiolucency in the right maxillary sinus, and cone beam computed tomography revealed a well‐defined hypodense lesion associated with Tooth 18 displaced into the sinus, causing cortical thinning. Following the removal of the tooth, an incisional biopsy was performed, and the specimen was sent for histopathological evaluation. Histopathology showed the presence of corrugated parakeratinized epithelium with a palisaded basal layer, confirming the diagnosis of OKC. Posteriorly, the patient was submitted for conservative treatment with curettage/enucleation followed by osteoplasty. No signal of recurrence was observed at the 2‐year follow‐up. This case reinforces the importance of differential diagnosis of radiolucent lesions of the maxillary sinus, as well as the conservative approach and the need for prolonged follow‐up given the possibility of recurrence.

## 1. Introduction

Odontogenic keratocyst (OKC) is a benign, slow‐growing odontogenic lesion, considered the third most common odontogenic cyst of the jaws [1]. OKC affects the posterior region of the mandible, causing bone reabsorption accompanied or not by root resorption and tooth displacement [2, 3]. Commonly, the progression of the cysts occurs in an anteroposterior direction, occupying the medullary spaces without provoking significant cortical expansion [4]. Because of the high rate of local recurrence and aggressiveness, the use of conservative therapeutic modalities (e.g., decompression and curettage/enucleation) along with long‐term follow‐up becomes mandatory for controlling and improving the quality of life of the patients [1, 3].

Historically, OKCs have been subject to reclassifications regarding their clinical behavior, with inclusion in the odontogenic tumor category by WHO (2005), and in accordance with the latest updates from the WHO (2017, 2022), the lesion was redefined as a cyst [5, 6]. Clinically, they present in two main forms: sporadic (nonsyndromic), occurring as a solitary lesion, or syndromic, showing multiple cysts associated with Gorlin–Goltz syndrome, whose pathogenesis in both cases involves genetic dysregulations in PTCH1 [6]. Despite OKC being a predominantly intraosseous lesion with prevalence in the mandible, observed in 65%–80% of affected individuals, some exceptional cases may develop in the maxilla, particularly in the maxillary sinus [7].

The occurrence of this cystic lesion in the maxillary sinus is rare, accounting for approximately 1% of all odontogenic cysts [2, 8]. As described in its intraosseous counterpart, OKC in the maxillary sinus may exhibit aggressive behavior, swelling, pain, and nasal obstruction [9, 10]. Because of the similarity to other pathologies that arise attached to the sinus membrane and the possibility of overlapping of anatomical structures, diagnosis is challenging [2]. Thus, we describe here a rare case of OKC involving the maxillary sinus, highlighting the importance of differential diagnosis with other radiolucent lesions and recognition of its clinical characteristics for effective decision‐making. This case report was prepared according to the SCARE 2025 guideline for surgical case reports [11].

## 2. Case Report

A 24‐year‐old male was referred to our Oral Surgery Service for surgical extraction of Tooth 18. Both extraoral and intraoral examinations yielded unremarkable results. The patient′s medical history did not indicate any abnormalities. A panoramic radiograph showed a well‐defined, extensive unilocular radiolucent area involving the right maxillary sinus (Figure 1A). Cone beam computed tomography (CBCT) scan revealed a hypodense image associated with Tooth 18, involving the maxillary sinus and resulting in cortical thinning, expansion, and disruption (Figure 1B–D). Despite these radiographic findings, during anamnesis the patient reported no previous episodes of infection or prior knowledge of any pathology involving the maxillary sinus. A provisional diagnostic hypothesis of odontogenic lesion and inflammatory conditions was made, including OKC, maxillary sinusitis and antral pseudocyst.

**Figure 1:**
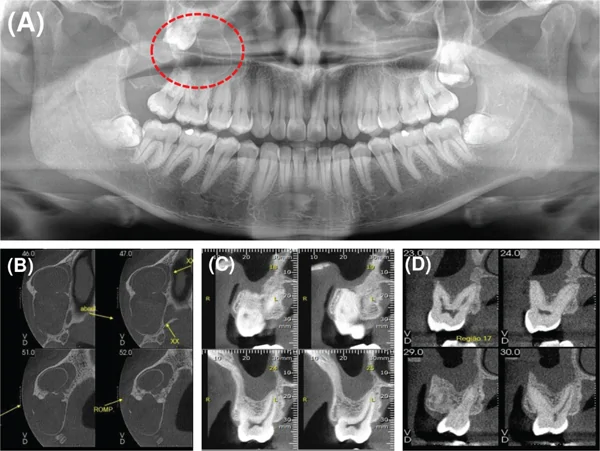
Imaging aspects of the maxillary sinus odontogenic keratocyst (OKC). (A) The panoramic radiograph exhibiting a radiolucent area in maxillary sinus (red circle). (B) Axial section CBCT revealed a well‐defined hypodense area, showing cortical displacement and perforation points of the lateral wall of the maxillary sinus. (C,D) Cross‐sectional images showing an area of opacification of the maxillary sinus in the region close to Tooth 17, absence of vestibular cortical bone and loss of bone tissue in the fenestration area.

Following the removal of the tooth, an incisional biopsy was performed in the area corresponding to the lesion for subsequent histopathological evaluation, which revealed a cystic cavity lined by corrugated parakeratinized stratified squamous epithelium, characterized by hyperchromatic basal cells arranged in a palisaded pattern and invasion of satellite cysts into the fibrous capsule (Figure 2 A,B). As a result, the diagnosis of maxillary sinus OKC was established. The patient underwent a conservative surgical excision of the lesion through curettage and enucleation followed by peripheral osteoplasty (Figure 3 A–D). Currently, he is being continuously monitored by the oral surgery team without showing signs of lesion recurrence at 2 years of follow‐up (Figure 4).

**Figure 2:**
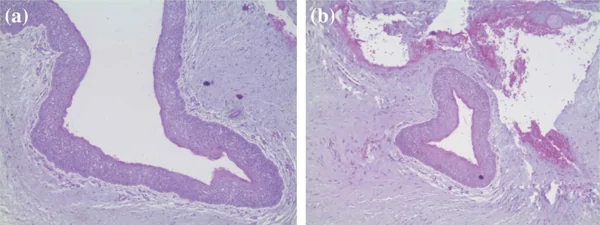
Histopathologic features of keratocystic odontogenic (OKC). (a) Photomicrograph showing epithelial lining composed of parakeratinized stratified squamous epithelium with a hyperchromatic basal cell layer (HE, original magnification 100x). (b) Presence of satellite cysts infiltrating the surrounding connective tissue (HE, original magnification 100x).

**Figure 3:**
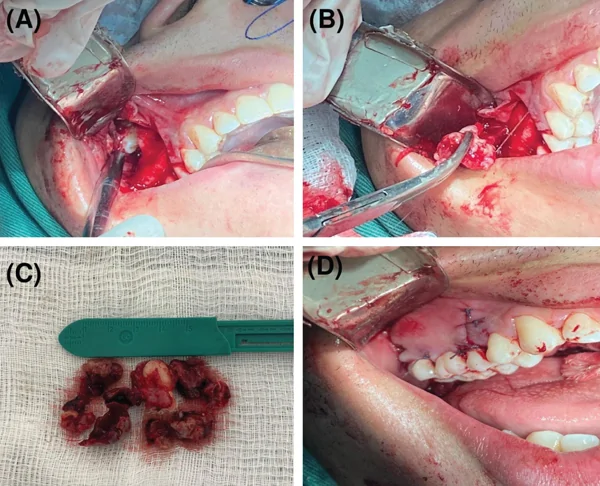
Surgical approach to the affected area. (A) Surgical access to the maxillary sinus and exposure of the impacted third molar. (B) Removal of the lesion from inside the maxillary sinus. (C) Fragment of the lesion associated with the impacted tooth. (D) Surgical closure and tissue readaptation through suturing.

**Figure 4:**
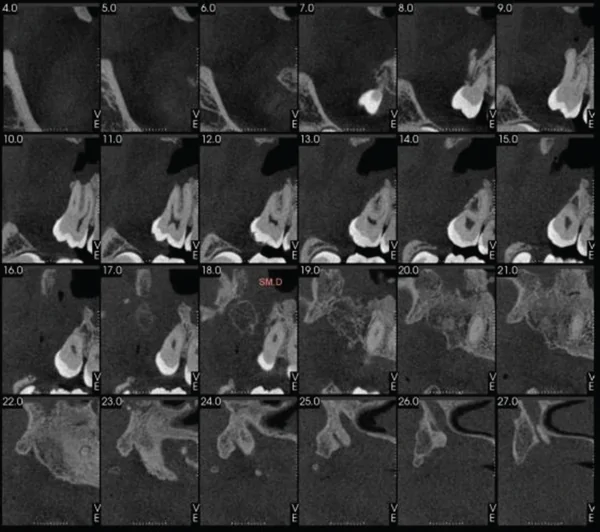
Imaging findings of the maxillary sinus 2 years after cyst enucleation surgery. Coronal CBCT sections revealed an area of new bone formation and absence of lesion recurrence.

## 3. Discussion

The maxillary sinus OKC is an exceptionally rare condition, initially identified by Cioffi et al. (1987) [12], accounting for approximately 1% of the total OKC cases mentioned in the literature [2, 8]. Typically, maxillary sinus OKCs are symptomatic and are often associated with dental anomalies such as unerupted, impacted, displaced, or incompletely formed teeth. In severe instances, the lesion can cause cortical expansion, displacement of the sinus floor, and invasion of the orbit and hard palate [4].

Furthermore, some patients might experience intraoral and extraoral maxillary swelling, erythema, or tenderness on palpation [9]. Because of the potential risk of damage to these anatomical structures, the conservative approach is the main treatment option, in contrast to more radical surgical interventions commonly seen for mandibular OKCs (e.g., chemical cauterization with Carnoy′s solution and peripheral osteotomy) [8, 13].

The etiology of OKC in maxillary sinus remains a subject of debate. Anatomically, the maxillary sinus represents the largest of the paranasal sinuses and is characterized as a pyramid‐shaped cavity [14]. Some studies support the hypothesis that the odontogenic epithelium becomes entrapped within the sinus during craniofacial development because of its anatomical proximity to the primitive oral cavity [7]. Conversely, maxillary sinus pathologies, particularly of odontogenic origin, may result from the disruption of the Schneiderian membrane [15]. The invasion and infiltration patterns of the maxillary sinus OKCs are generally attributed to the multicentric growth of cystic lesions, driven by the proliferation of localized epithelial cell aggregates [10]. In our case, however, the cyst exhibited a unilocular area with no evidence of multicellularity in the histopathological analysis.

Molecularly, the PTCH1 mutation has been described as the main genetic dysregulation observed in OKCs, especially in those cases associated with basal cell nevus carcinoma syndrome (also recognized as Gorlin–Goltz syndrome) [6]. This gene is responsible for encoding a transmembrane receptor that acts like a tumor suppressor by inhibiting the Sonic Hedgehog (SHH) signaling pathway, resulting in escaping apoptosis and increased cell proliferation [16]. Additionally, different studies have identified high expression in Ki‐67 and Bcl‐2 in cells of the basal layer of the epithelium, whereas the cyst wall shows intense activity of collagenolytic enzymes (e.g., collagenase) and pro‐inflammatory cytokines (e.g., IL‐1 and IL‐6) [17–20].

In turn, these factors contribute not only to rapid growth, but they also favor resorption and subsequent infiltration through destabilization of the bone matrix. Recently, Gonçalo et al. (2024) [21] and Uttagomol et al. (2025) [22], in their immunohistochemistry investigations, demonstrated a high expression of YAP in OKC epithelial cells, suggesting that activation of this biomarker may influence the aggressiveness of the cyst and its proliferation. Similarly, Pattnaik et al. (2024) [19] found immunopositivity for YAP in over 90% of neoplastic cells from evaluated ameloblastoma samples, highlighting its eventual role in the pathogenesis of the tumor. Therefore, these studies provide a molecule with a potential therapeutic target for improved clinical outcomes of aggressive odontogenic lesions, including OKC cases.

OKCs in the maxillary sinus present a unique clinical challenge. Unlike conventional OKCs, which have a high rate of recurrence up to 71%, there has been only one documented case of recurrence involving the maxillary sinus [13]. In general, the reported episodes of recurrence to mandibular OKCs are intrinsically linked to their biology, as a consequence of presence of friable capsule that is detachable from the overlying epithelial lining and infiltration of connective tissue by satellite microcysts, favoring the risk of these structures remaining in the surgical area [3]. Because of the topographic location of the maxillary sinus within the facial skeleton, it is not uncommon to observe lesions in the posterior maxilla, whether associated with a tooth or not, or lesions arising from periapical tissues that expand into this cavity [23, 24]. This pattern was observed in the present case, in which the cyst appears to have originated in the posterior maxilla with secondary involvement of the maxillary sinus.

Furthermore, the unique anatomical characteristics of the maxillary sinus, such as lower bone density of the maxilla, close proximity to tooth‐bearing structures, sinus membrane thickening, and pneumatization of the region, may create favorable conditions for the development of different pathologies [25–27]. For greater diagnostic accuracy in these cases, computed tomography (CT) is frequently recommended because of its superior ability to delineate lesion boundaries with precision, assess cortical expansion, identify the extension into adjacent soft tissues and establish its possible origin [10].

Although the maxillary sinus can be affected by odontogenic or fungal infections, the potential for cystic lesions should not be overlooked and included in the differential diagnosis [28]. Pathophysiologically, maxillary sinus OKC presents a rare and complex clinical challenge in terms of diagnosis and treatment [7]. Notoriously, the lesion′s potential to invade the bone cortex and instances of incomplete surgical removal observed in some cases necessitate the implementation of a more stringent follow‐up protocol [2].

## 4. Conclusion

In summary, radiolucent lesions can have various etiologies, including cysts, tumors, or infections. Accurate diagnosis is essential to determine the underlying cause and guide appropriate treatment. Untreated lesions can lead to complications such as cortical expansion, displacement of adjacent structures, or even invasion into surrounding tissues. Thus, early diagnosis of maxillary sinus OKC associated with adequate treatment can prevent these complications and provide a better quality of life for affected individuals.

## Funding

No funding was received for this manuscript.

## Ethics Statement

Ethical approval was not required for this single case report.

## Consent

Written informed consent was obtained from the patient to publish this report, including clinical photographs, in accordance with the journal′s patient consent policy.

## Conflicts of Interest

The authors declare no conflicts of interest.

## Supporting information


**Supporting Information** Additional supporting information can be found online in the Supporting Information section. File S1: SCARE Guideline Checklist 2025.

## Data Availability

The data that support the findings of this study are available from the corresponding author upon reasonable request.
